# Development of a Novel Simple Model to Predict Mortality in Patients With Systemic Lupus Erythematosus Admitted to the Intensive Care Unit

**DOI:** 10.3389/fmed.2021.689871

**Published:** 2021-07-22

**Authors:** Jinyan Guo, Zhen Huang, Maoxin Huang, Yujie He, Bing Han, Ning Ma, Zujiang Yu, Shengyun Liu, Zhigang Ren

**Affiliations:** ^1^Department of Rheumatology and Immunology, The First Affiliated Hospital of Zhengzhou University, Zhengzhou, China; ^2^Department of Cardiovascular Disease, The First Affiliated Hospital of Zhengzhou University, Zhengzhou, China; ^3^Department of Dermatology and Venereal Disease, The First Affiliated Hospital of Zhengzhou University, Zhengzhou, China; ^4^Department of Intensive Care Unit, The First Affiliated Hospital of Zhengzhou University, Zhengzhou, China; ^5^Department of Infectious Disease, The First Affiliated Hospital of Zhengzhou University, Zhengzhou, China

**Keywords:** systemic lupus erythematosus, intensive care unit, clinical feature, outcome, prognostic factors, prognostic model

## Abstract

**Background:** Patients with systemic lupus erythematosus (SLE) may sometimes require admission to the intensive care unit (ICU), and the outcome is poor. The aim of this study was to explore the clinical features of patients with SLE in the ICU, identify prognostic factors, and develop and evaluate a prognostic model to predict in-ICU mortality of patients with SLE.

**Patients and Methods:** This was a single center retrospective study in a tertiary medical institution in China. A total of 480 SLE patients with 505 ICU admissions from 2010 to 2019 were screened, and 391 patients were enrolled. The clinical feature and outcomes of the patients were analyzed. According to the random number table, patients were divided into two mutually exclusively groups named derivation (*n* = 293) and validation (*n* = 98). Prognostic factors were identified by a Cox model with Markov Chain Monte Carlo simulation and evaluated by latent analysis. The risk score was developed based on the derivation group and evaluated using the validation group.

**Results:** Among the 391 patients, 348 (89.0%) patients were females. The median age of patients was 34 years, and the median course of SLE was 6 months. The median APACHE II and SLEDAI were 17 and 10, respectively. The average in-ICU mortality was 53.4% (95% CI, 48.5–58.4%). A total of 186 patients were admitted to the ICU due to infection. Pneumonia (320/391, 81.8%) was the most common clinical manifestation, followed by renal disease (246/391, 62.9%). Nine prognostic factors were identified. The model had C statistic of 0.912 (95% CI, 0.889–0.948) and 0.807 (95% CI 0.703–0.889), with predictive range of 5.2–98.3% and 6.3–94.7% for the derivation and validation groups, respectively. Based on distribution of the risk score, 25.3, 49.5, and 25.2% of patients were stratified into the high, average, and low-risk groups, with corresponding in-ICU mortality of 0.937, 0.593, and 0.118, respectively.

**Conclusion:** Nine prognostic factors including age, white blood cell count, alanine transaminase, uric acid, intracranial infection, shock, intracranial hemorrhage, respiratory failure, and cyclosporin A/tacrolimus usage were identified. A prognostic model was developed and evaluated to predict in-ICU mortality of patients with SLE. These findings may help clinicians to prognostically stratify patients into different risk groups of in-ICU mortality, and provide patients with intensive and targeted management.

## Introduction

Systemic lupus erythematosus (SLE) is a chronic autoimmune disease of unknown etiology, which is characterized by a wide variety of auto-antibodies production and clinical manifestations (1). In the past few decades, the overall survival of patients with SLE has greatly improved and this may be ascribed to earlier diagnosis, immunosuppressive agent usage, and therapy of comorbidities (2). However, the outcome is still poor in patients with life-threatening medical conditions that need admission to the intensive care unit (ICU) (3, 4). Besides, in the last decades, SLE has become the most common disease in the ICU among various rheumatic diseases (5).

Currently, there are numerous reports regarding the clinical features, outcome, and prognostic factors of patients with SLE admitted to the ICU (6–17). However, the number of patients was relatively small in the majority of studies, and a scoring system was not established. There are several pre-existing scoring systems that have been used in the ICU, mainly including Acute Physiology and Chronic Health Evaluation II (APACHE II) and Simplified Acute Physiologic Score II (18, 19). The value of APACHE II in predicting outcome of patients with SLE admitted to the ICU have been assessed, and the results are somewhat contradictory (7–9, 11, 12, 17). Therefore, development of an effective prognostic model to predict in-ICU mortality of patients with SLE is urgently needed.

In this present study, we screened a total of 480 SLE patients with 505 ICU admissions spanning 9 years from 2010 to 2019, and ultimately 391 patients were enrolled. We explored the clinical features of the 391 patients and revealed that infection was the leading cause of ICU admission, as well as the most common clinical manifestation of patients in the ICU. We subsequently identified nine prognostic factors, and developed and evaluated a simple model to predict in-ICU mortality of patients with SLE. These results could be useful to prognostically stratify patients into different risk groups of in-ICU mortality, and provide patients with intensive and targeted management.

## Materials and Methods

### Patients and Study Design

**Figure 1:**
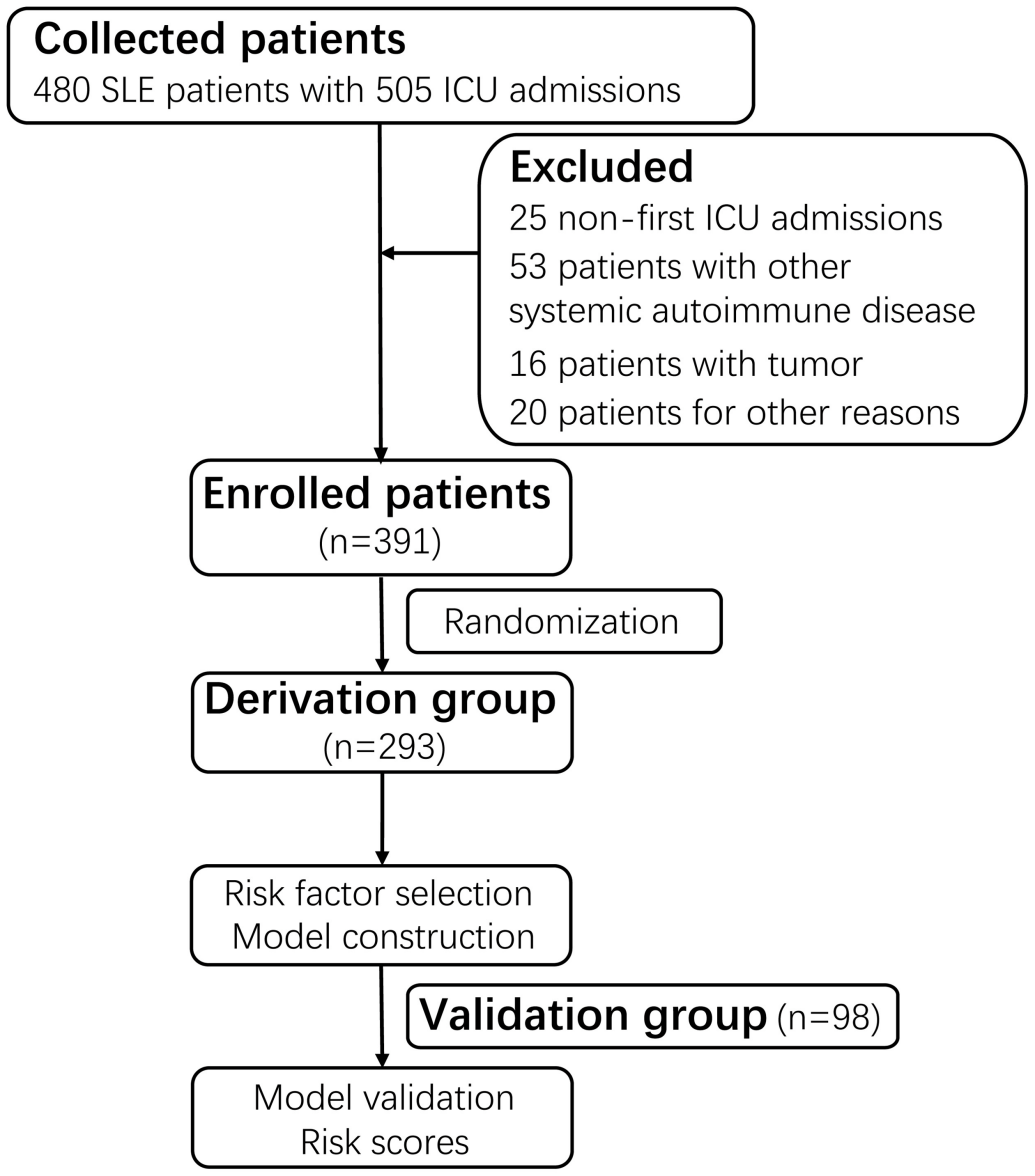
Flow diagram of the study. A total of 480 SLE patients with a combined 505 ICU admissions were screened. After excluding 25 non-first admissions, 53 patients with antiphospholipid syndrome or other systemic autoimmune disease, 16 patients with tumors, 20 patients admitted to the ICU for reasons unrelated with SLE, a total of 391 patients with the first ICU admission were enrolled. Based on the random number table, the 391 patients were divided into two mutually exclusive groups named derivation group (*n* = 293, 74.9%) and validation group (*n* = 98, 25.1%). The derivation group was used to identify prognostic factors for in-ICU survival, and the validation group was used for evaluation. SLE, systemic lupus erythematosus; ICU, intensive care unit.

Patients with SLE admitted to the ICU at the First Affiliated Hospital of Zhengzhou University between 1st October 2010 and 1st October 2019 were screened. The eligible patients for enrollment were 18 years or older and not pregnant. All patients fulfilled the American College of Rheumatology (ACR) criteria for the classification of SLE (20). In total, we identified 480 SLE patients with a combined 505 ICU admissions. Exclusion criteria included patients who were concomitant with antiphospholipid syndrome or other systemic autoimmune diseases (*n* = 53), or tumors (*n* = 16), or admission to the ICU for reasons unrelated with SLE (*n* = 20). We also excluded non-first ICU admissions (*n* = 25). According to the random number table, the final 391 unique patients were divided into two mutually exclusive groups named the derivation (*n* = 293, 74.9%) and validation (*n* = 98, 25.1%) groups. The derivation group was used to identify prognostic factors, whereas the validation group was employed for evaluation (Figure 1). Finally, this study was approved by the ethics committee of the First Affiliated Hospital of Zhengzhou University (No. 2019-KY-200) (Supplementary Material).

### Information Collection

Patients' information was collected detailedly from the electronic medical record. Clinically meaningful data (which were reliable, easily collected, and occurred at a frequency not <10 patients) was selected as candidate prognostic factors. The initial candidate prognostic factors included epidemiological data, clinical (ICU related and SLE related), and laboratory data. Systemic Lupus Erythematosus Disease Activity Index (SLEDAI) and APACHE II were documented within the first 24 h of ICU admission (18, 21). The APACHE II is composed of 12 routine physiologic measurements, age, and previous health status, which contains a score range of 0–71, with corresponding mortality range of 4–85% for non-surgical patients (18). Treatments including glucocorticoid (GC), hydroxychloroquine, cyclophosphamide, mycophenolate mofetil, and calcineurin inhibitors [CNIs, including cyclosporin A (CsA) and tacrolimus (Tac)] during ICU hospitalization were collected and treated as candidate prognostic factors.

### Definition of Terms

The diagnostic reasons for admission to the ICU were identified. A diagnosis of infection was made if patients had clinical features of infection accompanied with sufficient laboratory data and imaging findings to support the diagnosis. Renal disease referred to proteinuria >0.5 g/24 h, with or without renal insufficiency. Cardiovascular diseases were defined as any of the following conditions: cardiac arrest, acute myocardial infarction, or cardiac function class IV based on the New York Heart Association functional classification system (22). Neuropsychiatric lupus was classified according to the criteria of ACR (23). Hematologic disorder referred to thrombocytopenia, or leukemia, or anemia, or pancytopenia associated with SLE. Patients with acute thrombosis, or hemorrhage unrelated with thrombocytopenia, or other diagnoses were assigned to a single group named others.

### Primary Outcome Measure

In this study, the primary outcome was in-ICU death. For patients who were discharged from ICU against medical advice and died within 1 week, we also defined them as in-ICU death.

### Statistical Analysis

#### Prognostic Factor Selection and Evaluation

The derivation group with all candidate prognostic factors was used to fit a logistic model with Least Absolute Shrinkage and Selection Operator (LASSO) to sieve possible related factors with non-zero regression coefficients, otherwise, the factors were regarded insignificant and excluded from further analysis. Then, Markov Chain Monte Carlo (MCMC) simulation was employed to select the final prognostic factors, which have a positive coefficient in more than 95% or <5% of the simulations and hold a stable association with outcome. Subsequently, the prognostic factors selected by the MCMC simulation were used to fit a logistic model to the derivation group to develop the final prognostic model. Factors with missing values were imputed using multiple imputations with 10 imputations. The final imputed value was the mean of 10 imputations. Rates of missing value ranged from 3.8% (white blood cell, red blood cell, hemoglobin, platelet count, neutrophil percentage, and lymphocyte percentage), 4.8% (albumin and globulin), 5.8% (aspartate aminotransferase, urea, creatinine, uric acid (UA), and erythrocyte sedimentation rate), 6.6% (alanine transaminase (ALT), gamma-glutamyl transpeptidase, alkaline phosphatase, total bilirubin, direct bilirubin, and indirect bilirubin), 8.7% (prothrombin activity), 9.2% (lactic acid, complement C3, and complement C4), to 9.5% (C-reactive protein and PH).

The following indicators were calculated to assess the prognostic model performance: the Harrell C statistic to evaluate the overall predictive accuracy, the Hosmer-Lemeshow goodness-of-fit test to evaluate calibration, and the McFadden R square to evaluate explained variation. Receiver operating characteristic (ROC) curve and decision curve analysis (DCA) were performed to compare the prognostic model with APACHE II. In addition, discrimination was assessed among the observed outcomes in strata defined by quintiles of the predictive probabilities. We further divided patients in the derivation group into five mutually exclusive risk classes based on the quintiles, ranking the classes from the lowest risk (class 1) to the highest risk (class 5) for evaluation. We finally validated the prognostic model by comparing its performance in the derivation group with its performance in the validation group.

#### Risk Score

In this subsection, we constructed a simple risk score for each patient according to the regression coefficients estimated from the prognostic model with the derivation group, so as to facilitate the use of the prognostic factors and the prognostic model. Points for each prognostic factor were calculated by dividing the coefficient of the prognostic factor by summing the absolute value of coefficients in the model, multiplying by 100, and rounding to the nearest integer. We subsequently stratified patients into three risk groups following the distribution of the risk score, namely, low (<25th percentile), average (25th−75th percentile), and high (>75th percentile).

Analyses were implemented using SAS statistical software, version 9.4 (SAS institute lnc., Cary, NC). LASSO was performed using the glmnet package in R, version 3.6.3. In this study, we followed the Transparent Reporting of a Multivariable Prediction Model for Individual Prognosis or Diagnosis (TRIPOD) reporting guideline. Each of the 22 items of the TRIPOD statement was addressed.

## Results

### Patients' Characteristics

The general features of the 391 patients are summarized in Table 1. Among the 391 patients, 348 (89.0%) were female, the median age of patients and the median course of SLE were 34 years and 5 months, respectively. Then, the median values of platelet count (102 × 10^12^/L vs. 143.5 × 10^12^/L, *p* = 0.0021), globulin (28 vs. 31.6 g/L, *p* = 0.0010), and complement C4 (0.14 vs. 0.18 g/L, *p* = 0.0347) on ICU admission were lower in the derivation group compared to that in the validation group (Supplementary Table 1).

**Table 1:** General characteristics of the 391 patients.

**Characteristics**	**Total (*n* = 391)**	**Derivation (*n* = 293)**	**Validation (*n* = 98)**	***p*-values**
**Clinical features**				
Age, median (IQR) (years)	34 (26, 47)	33 (26, 48)	34 (27, 41)	0.2827
Female gender, *n* (%)	348 (89.0)	264 (90.1)	84 (85.7)	0.2294
Course of SLE, median (IQR) (month)	6 (0.5, 60)	5 (0.5, 60)	12 (0.6, 60)	0.5764
Length of stay before ICU, median (IQR) (day)	0 (0, 5)	0 (0, 6)	0 (0, 2)	0.2510
Length of stay in ICU, median (IQR) (day)	5 (2, 9)	5 (2, 9)	5 (2, 10)	0.9088
APACHEII, median (IQR)	17 (14, 24)	17 (14, 24)	17 (14, 22)	0.5147
SLEDAI, median (IQR)	10 (6, 15)	11 (6, 16)	9 (5, 14)	0.1560
**Therapies at the moment of ICU admission**				
Glucocorticoid, *n* (%)	285 (72.9)	220 (75.1)	65 (66.3)	0.1147
HCQ, *n* (%)	160 (40.9)	121 (41.3)	39 (39.8)	0.8135
CYC, *n* (%)	38 (9.7)	28 (9.5)	10 (10.2)	0.8451
MMF, *n* (%)	41 (10.5)	31 (10.6)	10 (10.2)	0.7067
CsA, *n* (%)	18 (4.6)	13 (4.4)	5 (5.1)	0.7831
Tac, *n* (%)	7 (1.8)	5 (1.7)	2 (2.0)	0.6853
**In-ICU mortality rate, % (95% CI)**	53.4 (48.5–58.4)	55.9 (50.3–61.7)	45.9 (36.1–55.8)	0.0841

*IQR, interquartile range; SLE, systemic lupus erythematosus; ICU, intensive care unit; APACHE II, Acute Physiology and Chronic Health Evaluation II; SLEDAI, Systemic Lupus Erythematosus Disease Activity Index. HCQ, hydroxychloroquine; CYC, cyclophosphamide; MMF, mycophenolate mofetil; CsA, cyclosporin A; Tac, tacrolimus; CI, confidence interval*.

A total of 209 patients died during ICU stay, comprising 164 and 45 patients in the derivation and validation groups, respectively. The average in-ICU mortality was 53.4% (95% CI, 48.5–58.4%). The in-ICU mortality for the derivation and validation groups were 55.9% (95% CI, 50.3–61.7%) and 45.9% (95% CI, 36.1–55.8%), respectively.

### Causes for Admission to the ICU

The causes for admission to the ICU of the 391 patients are depicted in Table 2. Infection was the leading cause of patients with SLE admission to the ICU. A total of 186 patients were admitted to the ICU due to infection, 159 of whom were with pneumonia. All the 57 patients admitted due to renal disease exhibited renal insufficiency and received renal replacement treatment during ICU stay. The remaining causes included neuropsychiatric disorders in 53 patients, cardiovascular diseases in 52 patients, hematologic disorders in 12 patients, and the other reasons in 31 patients.

**Table 2:** Primary causes of ICU admission of the 391 patients.

**Primary causes of ICU admission**	**Number of patients**
**Infection**	**186**
Pneumonia	159
Intracranial infection	11
Abdominal infection	8
Sepsis	6
Cellulitis of the neck	1
Acute laryngitis	1
**Renal disease**	**57**
Lupus nephritis	40
Acute renal injury	17
**Neuropsychiatric disorder**	**53**
Neuropsychiatric lupus	49
Hypoxic ischemic encephalopathy	1
Metabolic encephalopathy	1
Hypertensive encephalopathy	1
Coma caused by hyponatremia	1
**Cardiovascular disease**	**52**
Acute heart failure	39
Acute myocardial infarction	6
Pericardial effusion	4
Malignant arrhythmias	3
**Hematologic disorder**	**12**
Thrombocytopenia with active bleeding	6
Thrombotic Thrombocytopenic Purpura	3
Macrophage Activation Syndrome	2
Hemolytic anemia	1
**Other admission reasons**	**31**
Acute lupus pneumonitis	9
Diffuse alveolar hemorrhage	8
Acute hepatic failure	4
Acute pulmonary thromboembolism	3
Acute allergy reaction	3
Gastrointestinal hemorrhage	2
Bronchial artery hemorrhage	1
Acute pancreatitis	1

*ICU, intensive care unit*.

### Clinical Course and Treatment

The clinical characteristics of the 391 patients during ICU stay are summarized in Table 3. Pneumonia (320/391, 81.8%) was the most common clinical manifestation, followed by renal disease (246/391, 62.9%). Respiratory failure occurred in 216 patients. Of the 320 patients with pneumonia, microorganisms were identified in 70 patients by sputum culture. The most frequent microorganism was *Klebsiella pneumoniae* in 17 patients, followed by *Acinetobacter baumannii* in 14 patients, and *Pseudomonas aeruginosa* in 12 patients. Positive blood culture was detected in 14 patients, with *Listeria monocytogenes, Escherichia coli*, and *Staphylococcus aureus* the most common microorganisms (each in three patients). *Seventeen* patients developed intracranial infections, and 10 patients developed abdominal infections.

**Table 3:** Clinical feature during ICU of the 391 patients.

**Characteristics**	**Total *n* (%) (*n* = 391)**	**Derivation *n* (%) (*n* = 293)**	**Validation *n* (%) (*n* = 98)**	***p-*values**
**Cardiovascular disease**				
Pericarditis	116 (29.7)	93 (31.7)	23 (23.5)	0.1207
Heart failure	155 (39.6)	118 (40.3)	37 (37.8)	0.6591
AMI	11 (2.8)	8 (2.7)	3 (3.1)	0.8639
PAH	46 (11.8)	36 (12.3)	10 (10.2)	0.5796
**Respiratory system**				
Pleuritis	170 (43.5)	131 (44.7)	39 (39.8)	0.3956
DAH	13 (3.3)	10 (3.4)	3 (3.1)	0.8665
**Digestive system**				
Intestinal vasculitis	10 (2.6)	10 (3.4)	0 (0.0)	0.0639
Liver dysfunction	98 (25.1)	71 (24.2)	27 (27.6)	0.5116
Hypoproteinemia	184 (47.1)	140 (47.8)	44 (44.9)	0.6502
Seroperitoneum	73 (18.7)	55 (18.8)	18 (18.4)	0.9292
**Renal disease**	246 (62.9)	187 (63.8)	59 (60.2)	0.5209
Renal insufficiency	145 (37.1)	116 (39.6)	29 (29.6)	0.0761
**NPSLE**	55 (14.1)	40 (13.7)	15 (15.3)	0.6835
**Hematologic disease**				
Leukopenia	84 (21.5)	71 (24.2)	13 (13.3)	0.0221
Anemia	226 (57.8)	178 (60.8)	48 (49.0)	0.0411
Thrombocytopenia	163 (41.7)	134 (45.7)	29 (29.6)	0.0050
Pancytopenia	76 (19.4)	64 (21.8)	12 (12.2)	0.0377
MAS	13 (3.3)	11 (3.8)	2 (2.0)	0.4128
PE	10 (2.6)	8 (2.7)	2 (2.0)	0.7082
Cerebral infarction	14 (3.6)	11 (3.8)	3 (3.1)	0.7492
Intracerebral hemorrhage	14 (3.6)	9 (3.1)	5 (5.1)	0.6723
Gastrointestinal bleeding	29 (7.4)	23 (7.8)	6 (6.1)	0.5721
Thrombosis	29 (7.4)	25 (8.5)	4 (4.1)	0.1455
**Infection**				
Pneumonia	320 (81.8)	237 (80.9)	83 (84.7)	0.3975
Pulmonary fungal infection	91 (23.3)	77 (26.3)	14 (14.3)	0.0150
Respiratory failure	216 (55.2)	165 (56.3)	51 (52.0)	0.4615
Sepsis	14 (3.6)	10 (3.4)	4 (4.1)	0.7578
Intracranial infection	17 (4.3)	12 (4.1)	5 (5.1)	0.6723
Abdominal infection	10 (2.6)	10 (3.4)	0 (0.0)	0.0639
**Shock**	54 (13.8)	39 (13.3)	15 (15.3)	0.6201

*ICU, intensive care unit; AMI, acute myocardial infarction; PAH, pulmonary artery hypertension; DAH, diffuse alveolar hemorrhage; NPSLE, neuropsychiatric systemic lupus erythematosus; MAS, macrophage activation syndrome; PE, pulmonary embolism*.

Renal disease occurred in 246 patients, and 146 of whom developed renal insufficiency. Renal biopsy-confirmed lupus nephritis was identified in 72 patients, including class IV in 40 patients, class IV+V in 11 patients, class III in 10 patients, class III+V in seven patients, and class V in four patients. Moreover, gastrointestinal bleeding occurred in 29 patients, eight of whom had been treated with pulsed methylprednisolone prior. Intracranial hemorrhage occurred in 14 patients, eight of whom had thrombocytopenia before intracranial hemorrhage. Shock was developed in 54 patients, 43 of whom had infectious shock.

Management of the 391 patients during ICU is given in Table 4. Overall, 385 patients received GC, while 70 patients were treated with pulsed methylprednisolone. Hydroxychloroquine was used in 217 patients, cyclophosphamide in 34 patients, mycophenolate mofetil in 39 patients, and CNIs in 31 patients (including CsA in 26 patients and Tac in 5 patients), respectively. There were no differences between the two groups with respect to treatment.

**Table 4:** Management during ICU of the 391 patients.

**Management**	**Total, (*n* = 391) *n* (%)**	**Derivation, (*n* = 293) *n* (%)**	**Validation, (*n* = 98) *n* (%)**	***p-*values**
Mechanical ventilation	173(44.2)	134 (45.7)	39 (39.8)	0.3056
Renal replacement	87 (22.3)	72 (24.6)	15 (15.3)	0.0562
Inotropes	84 (21.5)	67 (22.9)	17 (17.3)	0.2494
Antimicrobial agents	336 (85.9)	253 (86.3)	83 (84.7)	0.6835
GC (prednisone 5–60 mg/d or equivalent)	385 (98.5)	289 (98.6)	96 (98.0)	0.6376
Pulsed MP (0.5–1.0 g/d)	70 (17.9)	58 (19.8)	12 (12.2)	0.0915
Plasmapheresis	23(5.9)	20 (6.8)	3 (3.1)	0.1703
Immunoglobulin (20–25 g/d)	131 (33.5)	96 (32.8)	35 (35.7)	0.5923
HCQ (0.2 bid)	217 (55.5)	161 (54.9)	56 (57.1)	0.7052
CYC (0.4–0.6 g/w)	34 (8.7)	27 (9.2)	7 (7.1)	0.5286
MMF (0.75 bid)	39 (10.0)	28 (9.6)	11 (11.2)	0.6333
CsA (180–240 mg/d)	26 (6.6)	23 (7.8)	3 (3.1)	0.0995
Tac (2–4 mg/d)	5 (1.3)	4 (1.4)	1 (1.0)	0.7926

*ICU, intensive care unit; GC, glucocorticoid; MP, methylprednisolone; HCQ, hydroxychloroquine; CYC, cyclophosphamide; MMF, mycophenolate mofetil; CsA, cyclosporin A; Tac, tacrolimus*.

### Prognostic Factors Selection and Evaluation

The LASSO simulation selected 17 candidate prognostic factors with non-zero coefficients taking the penalty parameter 0.02116773 (Table 5). On the basis, the MCMC method selected eight factors with a posterior probability of at least 0.95, including age, white blood cell count, ALT, UA, intracranial infection, shock, intracranial hemorrhage, and respiratory failure. For the convenience of calculations, ALT per 10, and UA per 100 were included in the final analysis. It is noteworthy that the value of CNIs was <0 for 95.6% of the cases in the 10,000 MCMC simulation, which indicated stable performance, so even the *P*-value was 0.081 in the final logistic model, it was included in the final model. The prognostic factors and prognostic model are shown in Table 6.

**Table 5:** Candidate prognostic factors selected by LASSO simulation.

**Number**	**Variables**	**Coefficients**
1	Intestinal vasculitis	−0.53805491
2	Liver dysfunction	0.10082777
3	Pancytopenia	0.04222531
4	Renal insufficiency	0.10824126
5	Respiratory failure	2.54588049
6	Intracranial infection	0.59310164
7	Intracerebral hemorrhage	0.73484724
8	Shock	0.83536019
9	Gastrointestinal bleeding	0.58261387
10	CNIs	−0.18745038
11	Age	0.13538059
12	WBC	−0.11250677
13	Plt	−0.01345163
14	ALT	0.16793747
15	Alb	−0.00432683
16	UA	0.20938012
17	PH	−0.14977542

*LASSO, Least Absolute Shrinkage and Selection Operator; CNIs, calcineurin inhibitors; WBC, white blood cell count; Plt, platelet count; ALT, alanine transaminase; Alb, albumin; UA, uric acid; PH, potential of hydrogen*.

**Table 6:** Prognostic model to predict in-ICU mortality based on derivation group.

**Prognostic factors**	**Regression coefficient**	**OR (95% CI)**	**Points**
Respiratory failure	2.54588049	34.00 (15.74–73.45)	29
Intracerebral hemorrhage	0.73484724	16.65 (2.25–123.15)	23
Shock	0.83536019	10.63 (1.89–59.77)	20
Intracranial infection	0.59310164	8.80 (1.41–54.83)	18
UA per 100	0.20938012	1.31 (1.09–1.58)	3
ALT per 10	0.16793747	1.07 (1.02–1.13)	1
Age	0.13538059	1.03 (1.00–1.05)	1
WBC	−0.11250677	0.92 (0.86–0.99)	−1
CNIs	−0.18745038	0.39 (0.13–1.13)	−8

*ICU, intensive care unit; OR, odds ratio; CI, confidence interval; UA, uric acid; ALT, alanine transaminase; WBC, white blood cell count; CNIs, calcineurin inhibitors*.

**Figure 2:**
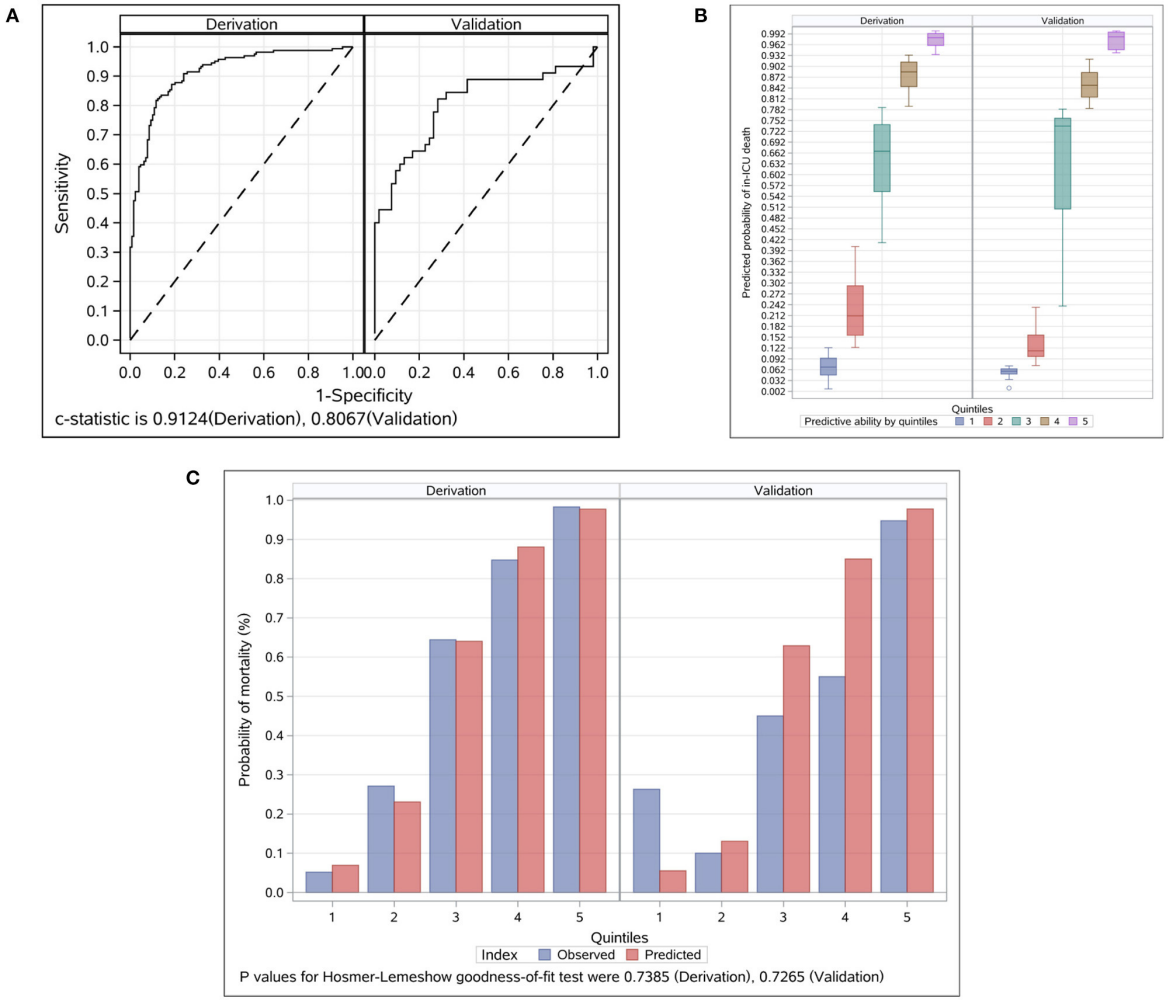
Evaluation for the final prognostic model in the derivation and validation groups. **(A)** ROC analyses for the evaluation of the risk model in the derivation and validation groups. Model performance in the validation group was comparable to that in the derivation group. The overall C Statistic was 0.912 (95% CI, 0.889–0.948) and 0.807 (95% CI 0.703–0.889) for the derivation and validation groups, respectively. **(B)** Probability of in-ICU death events by quintiles in the derivation and validation groups. Patients in each group were divided into five classes based on the nine prognostic factors. The mean observed in-ICU mortality ranges was from 5.2 to 98.3% for the derivation group, and from 6.3 to 94.7% for the validation group, respectively. **(C)** Observed vs. predicted values by quintiles in the derivation and validation groups. The *P*-value of the Hosmer and Lemeshow goodness-of-fit test was 0.7385 and 0.7265 for the derivation and validation groups, respectively. ICU, intensive care unit.

The prognostic model based on the nine prognostic factors was assessed in the derivation and validation groups, which reflected good discrimination, calibration, and fit. The overall Harrell C statistic was 0.9124 (95% CI, 0.889–0.948) and 0.8067 (95% CI, 0.703–0.889) for the derivation and validation groups, respectively (Figure 2A). The mean observed in-ICU mortality ranges was from 5.2% in the lowest predicted quintile to 98.3% in the highest predicted quintile for the derivation group, and from 6.3% in the lowest predicted quintile to 94.7% in the highest predictive quintile for the validation group, respectively (Figure 2B). The *P*-value of the Hosmer and Lemeshow goodness-of-fit test was 0.7385 and 0.7265 for the derivation and validation groups, respectively, suggesting that the model was well-fitted (Figure 2C). Notably, the explained variation was 0.4461 and 0.2382 for the derivation and validation groups, respectively, which met the proportional hazards assumption.

In addition, we compared the prediction efficiency of the model with that of APACHE II. ROC curve analyses displayed an area under the curve (AUC) of 0.796 and 0.735, for the prognostic model and APACHE II, respectively (Supplementary Figure 1A). On DCA, the prognostic model showed a higher threshold probability compared with APACHE II (Supplementary Figure 1B), which indicated a superior prediction efficiency of this model to APACHE II.

### Risk Score

The prognostic factor-specific points are shown in Table 6. The points ranged from 29 (respiratory failure) to −8 (CNIs). Respiratory failure, intracranial hemorrhage, shock, and intracranial infection were the top four prognostic factors. The score of each prognostic factor was obtained by multiplying the value of each factor by the corresponding point. The values of UA and ALT were obtained by dividing the actual levels by 100 and 10, respectively. The score of age was obtained by multiplying the age of the patient by one. Subsequently, the total score could be calculated by adding together each single item.

Scores varied slightly between the derivation and validation groups. The mean (SD) score was 67.1 (32.4) and 64.3 (39.6) for the derivation and validation groups, respectively. The risk stratification of in-ICU mortality is shown in Table 7. Based on the risk score, 25.3, 49.5, and 25.2% of patients in the derivation group were stratified into the high (total score ≥82.5+), average (total score 45.8–82.5), and low-risk groups (total score ≤ 45.8), with corresponding in-ICU mortality of 0.937, 0.593, and 0.118, respectively. The stratification of the validation group was not substantially different from that of the derivation group.

**Table 7:** Patient risk stratification based on risk score.

**Risk groups**	**Derivation group (*****n*** **=** **293)**		**Validation group (*****n*** **=** **98)**	
	**Patients, *n* (%)**	**In-ICU mortality, mean (*SD*)**	**Patients, *n* (%)**	**In-ICU mortality, mean (*SD*)**
High (82.5+)	74 (25.3)	0.937 (0.12)	19 (19.4)	0.935 (0.16)
Average (45.8–82.5)	145 (49.5)	0.593 (0.28)	47 (48.0)	0.667 (0.27)
Low (0–45.8)	74 (25.3)	0.118 (0.11)	32 (32.7)	0.085 (0.05)

*ICU, intensive care unit*.

## Discussion

There is evidence that the long-term survival rate of patients with SLE has improved significantly. The 5-year overall survival rate of SLE patients is 95%, and 10-year survival rate is now higher than 90% (24). Since last decades, SLE has become the most common systemic autoimmune disease in the ICU, accounting for 33.5% (5). However, the mortality rate of patients with SLE admitted in the ICU was still high, with in-ICU mortality as high as 28–78.5% (4, 7–12, 14, 15, 17). Many researches have been done to identify the prognostic factors associated with in-ICU mortality of patients with SLE, and the results were various among different studies. In the present study, we conducted a single center retrospective study based on 391 patients with SLE in the First Affiliated Hospital of Zhengzhou University, which is the largest tertiary medical hospital in China. We analyzed the clinical features of patients, identified nine risk factors, and developed and evaluated a prognostic model to predict in-ICU mortality of patient.

We found that infection was the leading cause of patients with SLE admission to the ICU, as well as the most common clinical manifestation of patients in the ICU. Patients with SLE have an increased risk of infection due to the intrinsic and extrinsic factors (25). The intrinsic factors mainly include the inherent dysfunctional immune system of SLE (26), and genetic factors (27). The administration of GC and immunosuppressant are the main extrinsic factors contributing to infection in patients with SLE (25, 28). Several studies have also shown that infection was the leading cause of patients with SLE admission to the ICU (6, 7, 9, 11, 12, 14, 15, 29). Besides, SLE disease activity and infection may deteriorate each other, which eventually lead to worse outcome. In clinical practice, it is necessary that clinicians should keep vigilance of infection in patients SLE at any time, so as to facilitate early diagnosis and treatment.

There were several pre-existing scoring systems to assess disease severity in patients admitted to the ICU. Among them, APACHE II is most widely used, and has been confirmed valuable in predicting in-hospital mortality among the general population (30). However, the value of APACHE II in predicting outcome of patient with SLE admitted to the ICU was inconsistent among different studies. Some studies found that APACHE II was one of the factors to predict in-ICU mortality in patients with SLE (7, 8, 11, 15), while the opposite results were found in other studies (9, 12, 17). Thus, it is necessary to develop a new prognostic model predicting in-ICU mortality of patients with SLE.

We conducted this study based on a large cohort of patients with SLE. We employed the MCMC algorithm to select prognostic factors following the marginal posterior probability that the factor should be in the model. Nine risk factors were identified, and a prognostic model was developed and evaluated in the derivation and validation groups, which reflected good discrimination, calibration, and fit. In addition, we compared the prognostic model with APACHE II, the results showed that the prediction efficiency of this model was superior to that of APACHE II. We further constructed a simple risk score system to stratify patients into three risk groups of in-ICU mortality. The in-ICU mortality rate was 93.7% in patient with risk score ≥82.5 and 59.3% in those with risk scores between 45.8–82.5, and 11.8% in patients with risk scores between 0 and 45.8, respectively. Through risk stratification, we found that 25.3% of the patients were at high-risk of in-ICU death, which emphasized the importance of identifying these patients on ICU admission, in order to provide patients with intensive and targeted management, and give patients and family members valuable information about prognosis.

In this study, the average in-ICU mortality rate was 53.4%, which was higher than that in most studies (7–9, 11, 14, 15). This may be attributed to the following reasons. In our study, nearly 63% of the patients had active renal disease, up to 82% the patients had infection, and some patients had serious complications such as gastrointestinal bleeding and intracranial hemorrhage, which may all account for the increased mortality. Besides, this may partly be limited to the level of medical treatment in our center.

Importantly, patients with other systemic autoimmune disease such as antineutrophil cytoplasmic antibody (ANCA) associated vasculitis may sometimes require admission to the ICU due to life threatening conditions (31–35). Compared with patients with SLE, patients with ANCA-associated vasculitis in the ICU were older, with a median age of more than 60 years, and were more common in male. The most common reason for admission to the ICU was active vasculitis rather than infectious disease. The overall in-ICU mortality rate of patients with ANCA-associated vasculitis was comparatively lower than that in SLE, mostly between 11 and 33.3%, with the highest being 58.5%. Besides, patients with Adult-onset still disease (AOSD) may occasionally admit to the ICU because of vital organ involvements, with the most common organ manifestation being respiratory failure, followed by cardiocirculatory failure and macrophage activation syndrome (36). One study analyzed the clinical feature of 20 patients with AOSD in the ICU (36), and two patients died in the ICU. Compared with patients with SLE, patients with AOSD were more prone to admit to the ICU at disease onset, and more likely to develop macrophage activation syndrome.

There were several limitations of this study. Firstly, this was a single center study involving only Chinese population, which limited the validity of the results. Secondly, given the retrospective nature of the observation, some informative data like previous treatment prior to ICU admission and SLE-related organ damage were lack, thus their possible association with in-ICU mortality could not be tested. Thirdly, the belimumab, a monoclonal antibody binding to soluble human B-lymphocyte stimulator used in patients with SLE, was approved in China in the year 2019. None of the patients in our study received belimumab, thus the possible influence of this agent on patients' prognosis could not be evaluated. Fourthly, the study primarily focused on prognostic factors of in-ICU mortality, but lacked of elements of novelty in terms of suggestions in the management of SLE patients. Fifthly, there was a lack of external validity of this study, and the prognostic model was not compared to APACHE II as well. Finally, more research is needed to estimate the generality of our results.

In summary, in this study, we analyzed the clinical features and outcome of patients with SLE in the ICU in a single center in China. The average in-ICU mortality was 53.4% (95% CI, 48.5–58.4%). Infection was the leading cause of patients with SLE admission to the ICU, as well as the most common clinical manifestation of patients in the ICU. Nine prognostic factors including age, white blood cell count, ALT, UA, intracranial infection, shock, intracranial hemorrhage, respiratory failure, and CsA/Tac usage were identified. A simple model was developed and evaluated to predict in-ICU mortality of SLE patients. These findings may help clinicians to prognostically stratify patients into different risk groups of in-ICU mortality, and provide patients with intensive and targeted management.

## Data Availability Statement

The original contributions presented in the study are included in the article/Supplementary Material, further inquiries can be directed to the corresponding author/s.

## Ethics Statement

The studies involving human participants were reviewed and approved by the ethics committee of the First Affiliated Hospital of Zhengzhou University. The ethics committee waived the requirement of written informed consent for participation.

## Author Contributions

JG, ZH, ZR, and SL designed the study. JG, MH, YH, BH, NM, and ZY collected clinical data. JG, ZH, ZR, SL, and MH analyzed the data. JG, ZH, and ZR wrote the manuscript with contributions from all authors. All authors reviewed and approved the submitted version.

## Conflict of Interest

The authors declare that the research was conducted in the absence of any commercial or financial relationships that could be construed as a potential conflict of interest.

## Supplementary Material

Supplementary Figure 1 Comparison of the prognostic model with APACHE II.

Supplementary Table 1 Laboratory data on ICU admission of the 391 patients.
