# Supplementary figures and images for: Development of a Novel Simple Model to Predict Mortality in Patients With Systemic Lupus Erythematosus Admitted to the Intensive Care Unit
Source: Front Med (Lausanne). 2021 Jul 22;8:689871. doi: 10.3389/fmed.2021.689871 (PMC8339434; doi:10.3389/fmed.2021.689871)

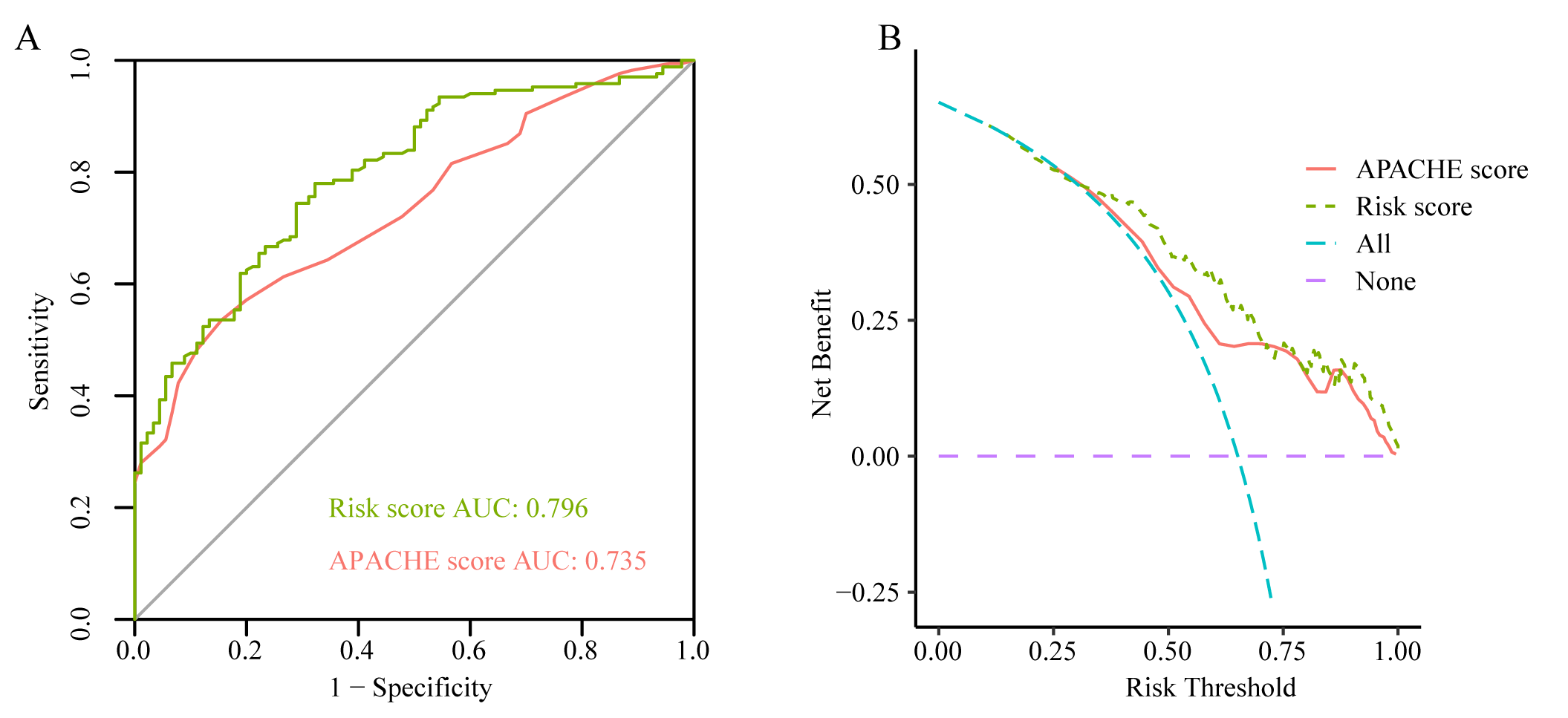

Supplement: Supplementary Figure 1 — Comparison of the prognostic model with APACHE II. [file Image_1.TIF]
