# Supplementary material for: Development of a Novel Simple Model to Predict Mortality in Patients With Systemic Lupus Erythematosus Admitted to the Intensive Care Unit
Source: Front Med (Lausanne). 2021 Jul 22;8:689871. doi: 10.3389/fmed.2021.689871 (PMC8339434; doi:10.3389/fmed.2021.689871)
Supplement: Supplementary Table 1 — Laboratory data on ICU admission of the 391 patients. [file Table_1.PDF]

Table S1 Laboratory data on ICU admission of the 391 patients

Parameters	Total, median (IQR) (n=391)	Derivation median (IQR) (n=293)	Validation median (IQR) (n=98)	*p* values
WBC (3.5-9.5×10^9/L)	7.1 (4.1, 0.9)	7.2 (4, 10.5)	7 (4.2, 12.4)	0.2505
RBC (3.8-5.1×10^{12}/L)	3.3 (2.6, 3.9)	3.2 (2.6, 3.9)	3.3 (2.7, 4.1)	0.2347
Hb (115-150 g/L)	93 (77, 116)	92 (76, 114)	94 (80, 118)	0.5090
Plt (125-350×10^{12}/L)	113 (61, 184)	102 (54, 173)	143.5 (81, 201)	0.0021
NE% (40-75%)	83.1 (73.2, 89.9)	82.3 (72.3, 89.7)	84.5 (77.3, 90.5)	0.1028
Lyn% (20-50%)	10.6 (6.2, 17.4)	10.9 (6.2, 18)	9.8 (6.6, 15.2)	0.3138
Alb (35-55 g/L)	27.5 (22.8, 32.5)	27.3 (22.1, 32.5)	27.7 (24.2, 32.5)	0.3013
Glb (20-35 g/L)	28.7 (24, 35.1)	28 (23, 34.6)	31.6 (27.4, 36.7)	0.0010
ALT (0-40 U/L)	28 (15, 65)	28 (16.9, 68)	26.5 (15, 45)	0.2566
AST (0-40 U/L)	34 (21, 77)	35 (22, 79)	33.5 (20, 63)	0.4475
GGT (0-58 U/L)	44 (23, 120)	45 (22.7, 121.4)	43.2 (24, 108.8)	0.9695
ALP (35-105 U/L)	72 (52, 108)	70 (52, 107)	75 (52.9, 109.5)	0.4132
TBil (0-25 mmol/L)	7.9 (5, 17.5)	7.9 (4.9, 17.5)	7.8 (5.1, 17)	0.6698
DBil (0-10 mmol/L)	3.8 (2.4, 8.4)	3.7 (2.3, 8.2)	4.1 (2.6, 8.4)	0.4698
IBil (0-14 mmol/L)	3.8 (2.2, 6.9)	3.8 (2.3, 6.5)	3.5 (2.1, 7.3)	0.9333
PTa (70-150 s)	110 (87, 132)	110 (88.1, 131.4)	109.5 (75.1, 132.8)	0.4485
Urea (2.2-8.2 mmol/L)	8.5 (5.4, 16.4)	8.5 (5.6, 16.7)	8.6 (5, 14.7)	0.4874
Cr (20-115 μmol/L)	75.8 (49, 158)	77 (50, 169)	70 (48, 140)	0.4399
UA (140-360 mmol/L)	330 (220, 479)	340 (225, 480)	296 (206, 466)	0.3001
CRP (0-10 g/L)	29.4 (7.3, 79.8)	27.3 (6.5, 75.2)	45.1 (11, 84.1)	0.0556
ESR (0-15 mm/h)	37 (19, 79)	36 (19, 69.25)	45 (19.5, 101)	0.1916
PH (7.35-7.45)	7.4 (7.4, 7.5)	7.4 (7.4, 7.5)	7.4 (7.4, 7.5)	0.9893
LA (0.5-1.7 mmol/L)	1.6 (1.0, 2.8)	1.6 (0.9, 2.9)	1.6 (1.025, 2.675)	0.8146
C3 (0.9-1.8 g/L)	0.64 (0.41, 0.88)	0.61 (0.38, 0.85)	0.75 (0.43, 0.99)	0.0740
C4 (0.1-0.4 g/L)	0.15 (0.09, 0.22)	0.14 (0.09, 0.21)	0.18 (0.10, 0.25)	0.0347
Anti-dsDNA (0-100 U/L)	123 (56, 450)	123.5 (60, 432.8)	99 (56, 456)	0.8160

ICU, intensive care unit; IQR, interquartile range; WBC, white blood cell count; RBC, red blood cell count; Hb, hemoglobin; Plt, platelet count; NE%, neutrophil percentage; Lyn%, lymphocyte percentage; Alb, albumin; Glb, globulin; ALT, alanine transaminase; AST, aspartate aminotransferase; GGT, gamma-glutamyl transpeptidase; ALP, alkaline phosphatase; TBil, total bilirubin; DBil, direct bilirubin; IBil, indirect bilirubin; PTa, prothrombin activity; Urea, blood urea nitrogen; Cr, creatinine; UA, uric acid; CRP, C-reactive protein; ESR, erythrocyte sedimentation rate; PH, potential of hydrogen; LA, lactic acid; C3, complement C3; C4, complement C4.
